# Bone Densitometry Parameters in Females with Ehlers-Danlos Syndrome—Does the Hypermobile Subtype Increase the Risk of Low Bone Mass in Patients with Ehlers-Danlos Syndrome?

**DOI:** 10.3390/jcm14030941

**Published:** 2025-02-01

**Authors:** Bernadetta Kałuża, Ivan Rychlik, Jan Domański, Aleksandra Żuk-Łapan, Emilia Babula, Iga Poprawa, Jakub Podstawka, Ewa Kowalów, Edward Franek

**Affiliations:** 1 Department of Internal Medicine, Endocrinology and Diabetology, National Medical Institute of the Ministry of the Interior and Administration, 02-507 Warsaw, Poland; 2 Students Scientific Group of the Medical University of Warsaw at the Department of Internal Medicine, Endocrinology and Diabetology, National Medical Institute of the Ministry of the Interior and Administration, 02-091 Warsaw, Poland; 3 Department of Internal Medicine, Third Faculty of Medicine, Charles University, and Královské Vinohrady University Hospital, 100 00 Prague, Czech Republic; 4 Department of Human Epigenetics, Mossakowski Medical Research Institute, Polish Academy of Sciences, 02-106 Warsaw, Poland

**Keywords:** Ehlers-Danlos syndrome, bone densitometry, low bone mass

## Abstract

**Background:** The purpose of this study was to assess bone densitometry parameters in patients with classical and hypermobile Ehlers-Danlos syndrome (EDS) and to determine whether the hypermobile subtype increases the risk of low bone mass, which is particularly important in this patient group, since the genetic mutation responsible for this subtype is still unknown. **Material and Methods:** In order to conduct this study, we collaborated with the EDS society in Poland. A total of 30 females of reproductive age who were included in the study were divided into two groups: Group 1—those with classical EDS (*n* = 9) and Group 2—those with hypermobile EDS (*n* = 21). Routine laboratory test results, bone turnover markers, and densitometry parameters were evaluated and compared. **Results:** The study groups showed no differences in terms of densitometry parameters or markers of calcium-phosphate metabolism. A multivariate logistic analysis demonstrated no increase in the risk of low bone mass (defined as a Z-score lower than −2) in patients with hypermobile EDS (OR 0.067 [95% Cl 0.0–20.927]; *p* = 0.356). **Conclusions:** The hypermobile subtype of EDS does not increase the risk of low bone mass; there were no significant differences between patients with hypermobile EDS and those with classical EDS in terms of either densitometry parameters or markers of calcium-phosphate metabolism. Although patients with hypermobile EDS are not at a higher risk of developing low bone mineral density, they should be regularly monitored for any calcium and phosphate metabolism abnormalities.

## 1. Introduction

Ehlers-Danlos syndrome (EDS) is a group of genetic disorders with an autosomal dominant or autosomal recessive inheritance pattern or resulting from de novo mutations of genes responsible for the synthesis of collagen and other connective tissue components. EDS may manifest primarily with joint hypermobility and excessive skin elasticity; however, many other abnormalities have also been described, including cardiovascular anomalies (such as aortic aneurysm, mitral valve prolapse, arrhythmias, and Raynaud fenomenon). Currently, there are 13 known subtypes of EDS, with the causative mutation remaining unknown only in the case of the hypermobility subtype [1,2,3]. Despite limited data about the epidemiology of EDS, the total prevalence of the disease depends on its subtype. The hypermobile subtype is the most common one, affecting 80–90% of all patients with EDS. It affects an estimated 1–5/10,000 individuals [4]. More common are hypermobility spectrum disorders and symptomatic joint hypermobility syndromes, with a reported prevalence of 1 in 500 people, most of whom (70%) are women [5]. Due to the ubiquitous nature of collagen and other connective tissue components, patients with EDS exhibit multiple comorbidities that may affect any system in the body. Bone disorders are more common in patients with EDS; however, their rates vary between the individual subtypes. The main musculoskeletal complications in patients with hypermobile EDS are joint hypermobility and instability, with early complications, such as soft tissue lesions, sprains, dislocations, or subluxations, and late complications, such as chronic joint pain, osteoarthritis, muscle tension and spasm, muscle and ligament tears, tendon ruptures, and tendonitis [6,7].

Individual subtypes of EDS show differences in the patients’ bone mineral density (BMD). This may be due to the different underlying genetic mutations, particularly if the mutation affects a gene affecting osteocyte function, such as in the case of spondyldysplastic EDS, which is associated with the zinc transporter-encoding *SLC39A1* gene, whose mutation disrupts osteoblast maturation and reduces both trabecular and cortical bone mass [8]. Bone mass may also become lower as a result of decreased type I collagen expression, which is caused by a *PRDM5* mutation [9]. The effects of EDS on BMD may also be indirect. For example, neurological abnormalities or increased ligament elasticity cause the forces generated by muscles to be transferred onto bones less efficiently, which adversely affects bone structure [10,11].

Higher rates of vertebral fractures have been reported in adult patients with hypermobile EDS, whether or not their BMD was low or normal [12,13,14]. Nonetheless, the data are inconsistent, and it is not certain whether hypermobile EDS is associated with lower BMD.

The purpose of this study was to assess bone densitometry parameters in women with hypermobile and classical EDS and to determine if the hypermobile subtype increases the risk of low bone mass. This has not been definitively determined in previous studies; however, this is particularly important in terms of this population’s characteristics and the future search for the genetic mutation responsible for the hypermobile subtype of EDS.

## 2. Material and Methods

We reached out to the EDS society in Poland to collaborate on patient recruitment for our study. We posted a questionnaire with information about this study on the EDS society’s Polish website and then contacted those patients who expressed an interest in participation. Those who met the study’s inclusion criteria and did not meet any of the exclusion criteria were included in our study.

The study’s inclusion criteria were as follows: female sex, age over 18 years, and a diagnosis of the hypermobile or classical subtype of EDS based on the current criteria. The hypermobile subtype is diagnosed when three criteria are met: criterion 1—generalized joint hypermobility; criterion 2—two or more of the following features: A. systemic abnormalities in connective tissue structure, B. one or more first-degree relatives meeting the diagnostic criteria for hypermobile EDS, and C. musculoskeletal symptoms; and criterion 3—all of the following prerequisites must be met: 1. absence of unusual skin fragility, which might suggest other types of EDS, 2. exclusion of other hereditary or acquired connective tissue disorders, and 3. exclusion of alternative diagnoses that may also include hypermobility. The classical subtype is diagnosed based on skin hyperextensibility; abnormal scaring; COL5A1, COL5A2, and COL1A1 gene mutations; and autosomal dominant inheritance [15,16,17].

The study’s exclusion criteria were a lack of informed consent; pregnancy; postmenopausal status; the use of contraceptives, hormone replacement therapy, or systemic steroid therapy; and chronic kidney disease stage G3a or higher (eGFR < 60 mL/min/1.73 m^2^).

Ultimately, out of the 230 patients who expressed an interest in study participation, 30 patients were included in this study. The patients were divided into two groups, depending on their EDS subtype. Group 1 (*n* = 9) comprised patients with classical-like EDS, and Group 2 (*n* = 21) comprised patients with hypermobile EDS.

This study was approved by the ethics committee at the Medical University of Warsaw (approval No. KB/106/2023 of 3 July 2023). Each patient signed a written informed consent form. This study was conducted according to the current standards set forth in the World Medical Association’s Declaration of Helsinki and Good Clinical Practice.

Medical history and a morning blood sample under fasting were obtained from each patient. The blood tests included total calcium, inorganic phosphate, parathormone, 25-OH vitamin D, bone-specific alkaline phosphatase, beta-CrossLaps (CTx), osteocalcin, sodium, potassium, thyroid-stimulating hormone, dehydroepiandrosterone sulfate (DHEA-S), and cortisol levels.

Each patient underwent left femoral and lumbar spine densitometry (GE Healthcare Lunar Prodigy bone densitometer), including bone mineral density (in g/cm^2^) and—due to the young age of the study participants and the age difference between the groups—an age-matched Z-score for the evaluated region, expressed as a percentage in order to diagnose osteoporosis. We analyzed the regions of interest in the left femur, and these included total neck, upper neck, lower neck, Ward’s triangle, a trochanter, a shaft, and total femur, and the analyzed regions of the lumbar spine included vertebrae L1, L2, L3, and L4 and L1–L2, L1–L3, L1–L4, L2–L3, and L3–L4.

Statistical analysis was conducted with Statistica^®^ software (13.3). Statistical significance was determined with the use of tests suitable for the nature of the data and the type of data distribution (Student’s *t*-test, Mann–Whitney U test, or chi-square test). A multivariate logistic regression model was used to determine the statistical significance of the evaluated variables in predicting hormonal disorders.

## 3. Results

**Figure 1:**
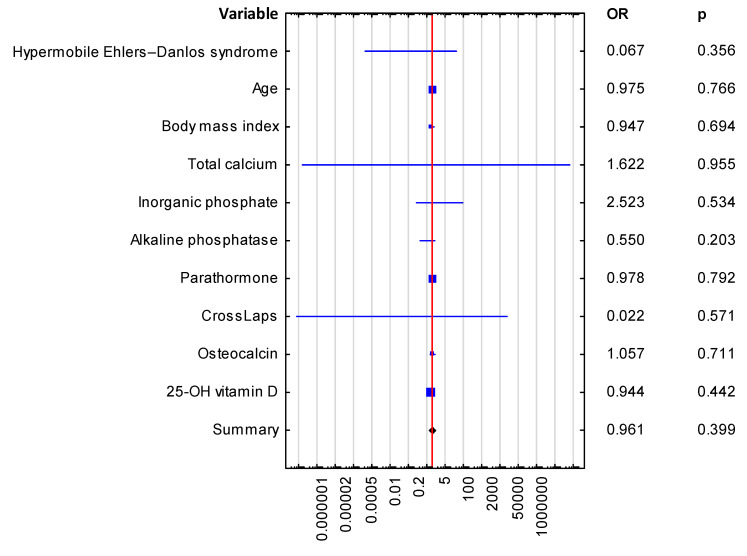
Multivariate logistic regression in the assessment of the predictors of a Z-score below −2.0.

Out of the 30 patients (21 of whom had hypermobile EDS and 9 had classical EDS) 17 patients (56.7%) reported a history of fractures, with 15 cases of upper limb fractures and 2 cases of lower limb fractures. A total of six patients had a Z-score below −2.0 (three with hypermobile EDS and three with classical EDS). The Z-score was below −2 in the following regions of the left femur: the upper neck in two cases, Ward’s triangle in three cases, the trochanter in four cases, and the total neck in one case. The evaluated groups did not differ significantly in terms of BMD at the following sites: the left femur neck, the left femur upper neck, the left femur lower neck, the left femur Ward’s triangle, the left femur trochanter, the left femur shaft, L1–L4, L1, L2, L3, L4, L1–L2, L1–L3, L2-L3, L2–L4, or L3–L4; however, we observed that the age-matched BMD of the left femur Ward’s triangle (88.111 ± 10.925 vs. 88.286 ± 15.153 [%]) and the left femur trochanter (86.222 ± 12.143 vs. 85.333 ± 12.055 [%]) was lower than the age-matched BMD of the lumbar spine (L1–L4 age-matched BMD [%]: 101.333 ± 14.361 vs. 104.1 ± 10.004). Eighteen patients, most of whom were from the classical EDS group, were found to be vitamin D3-deficient. The mean Vitamin D concentration was significantly lower in the classical EDS group. They were also older (39.8 ± 8.3 years vs. 33.5 ± 9.0 years, *p* = 0.045). In addition to the above, there were no differences between the groups in terms of bone density parameters or markers of calcium-phosphate metabolism (Table 1, Table 2). A multivariate logistic regression analysis demonstrated no evidence of a higher risk of osteoporosis in patients with hypermobility EDS (Table 3, Figure 1).

## 4. Discussion

Despite the fact that the prevalence of any kind of fractures has been reported to be higher in hypermobility EDS, our study showed no differences in terms of bone densitometry parameters, markers of bone turnover, or markers of calcium-phosphate metabolism between the study groups. The multivariate logistic regression analysis demonstrated no evidence that hypermobile EDS increases the risk of low bone mass, expressed as a Z-score below −2.

Like the classical subtype of EDS, the hypermobile subtype does not increase the risk of osteoporosis. Studies in patients with hypermobile EDS have shown slightly reduced bone mineral density, without a definitive increase in fracture rates, which remains controversial [3]. A study comparing patients with (hypermobile and classical) EDS and those from an age- and sex-matched control group showed that females with EDS had lower femoral neck and lumbar spine BMD by 0.9 SD and 0.7 SD, respectively, and had a higher rate of low-trauma fractures [3]. Conversely, another study showed no differences in fracture rates while demonstrating lower femoral neck BMD in patients with EDS, which may have been associated with decreased exercise levels [18]. Another study comparing females with hypermobile EDS or hypermobility spectrum disorder with sex- and age-matched controls showed the former to have a lower cortical thickness in the tibial diaphysis and a lower muscle mass while having comparable lumbar spine, total body, and trabecular and cortical volumetric BMD values in the tibia [19]. Patients with classical and hypermobile EDS subtypes were shown to have vertebral shape abnormalities [20]. Studies have shown that patients with EDS are more likely to have a 20% or more greater difference between the anterior, middle, and posterior heights of vertebrae, which is not necessarily associated with fractures but rather with dysplastic changes that occur during bone development [12,14]. However, an increased risk of osteoporosis combined with vertebral shape abnormalities were observed in patients with classical-like, kyphoscoliotic, arthrochalasia, and spondylodysplastic subtypes of EDS [20]. The risk factors for developing osteoporosis in these patients, apart from genetic defects and age, seem to be reduced muscle contractility, diminished transfer of forces from muscle to bone (which may be due to abnormalities in tendon structure and function), and limited physical activity due to pain [20,21,22].

The genetic mutation causing the hypermobile subtype of EDS has not been identified. However, although mutations of the genes encoding the proteins responsible for post-translational collagen modifications, transcriptional regulators, extracellular matrix proteins, enzymes involved in proteoglycan synthesis, mediators of the innate immune response, and zinc transporters have been detected in patients with other EDS subtypes, the potential effect of these mutations on bones have not been well identified [20]. Nonetheless, EDS may affect bone structure via a number of factors associated with other affected organs or systems and via lifestyle modifications and physical activity, the last of which may be limited by pain [23,24]. The gait of patients with classical and hypermobile EDS was demonstrated to be slower and the step length shorter, which affects coordination of movements, maintenance of balance, muscle strength, and, consequently, the risk of falls and fractures [25]. Patients with classical and hypermobile EDS have been reported to have decreased tendon stiffness [10,11]. The altered transmission of forces from muscle to bone via structurally altered tendons may result in secondary bone weakening, as well as less effective muscle contraction, particularly when tendon stiffness is low [26].

Patients with hypermobile EDS are known to primarily exhibit joint hypermobility and instability, which are associated with a loosened structure of ligaments, joint capsules, and tendons [21]. Joint instability is a risk factor of subluxations, dislocations, and soft tissue lesions, such as ganglion cysts, molluscoid pseudotumors, spheroids, or piezogenic papules, which may ultimately lead to tendon inflammation and rupture, increased muscle tension, osteoarthritis, and chronic pain syndrome [27,28,29,30,31,32]. Joint hypermobility is the main feature assessed with the Beighton scoring system to diagnose EDS, including the hypermobile subtype [6,33,34,35]. All these parameters working together may explain the increased risk of fractures; however, they may have no effect on the development of osteoporosis, which may be age-dependent too [3]. Adults with hypermobile and classical EDS have been observed to have an increased risk of vertebral and overall fractures, despite normal bone mass density [12,13,14].

Limitations: the most important limitation is the small population; however, this is due to the fact that EDS is a rare condition, and the study group is very homogeneous.

Future direction: Prospective studies should be carried out for a large group of patients, with long follow-up periods and an assessment of other EDS subtypes whose genetic mutations are documented, in order to evaluate how factors, such as physical activity, lifestyle, tendon and muscle dysfunction, joint hypermobility, and bone vulnerability, as well as specific genetic mutations, might affect bone metabolism and predisposition to osteoporosis and fractures. Such studies might contribute to developing treatment standards and strategies for patients with EDS and help identify the gene whose mutation is responsible for hypermobile EDS.

## 5. Conclusions

Patients with hypermobile EDS are characterized by similar bone mass to those with classical EDS. They show no significant differences from patients with classical EDS in either parameters associated with bone densitometry or markers of calcium-phosphate metabolism, with the exception of a higher vitamin D level. Although patients with hypermobile EDS are not at a higher risk of developing low BMD, they should be regularly monitored for any calcium and phosphate metabolism abnormalities.

## Figures and Tables

**Table 1:** Patient characteristics—clinical characteristics, femoral densitometry, lumbar spine densitometry, and laboratory tests. Group 1—patients with classical Ehlers-Danlos syndrome; Group 2—patients with hypermobile Ehlers-Danlos syndrome.

Characteristics	Group 1 (N = 9)	Group 2 (N = 21)	Significance of the Difference (*p*-Value)
Left femur neck BMD [g/cm^2^]	0.919 ± 0.133	0.947 ± 0.118	0.824
Left femur neck age-matched BMD [%]	92.875 ± 13.432	94 ± 11.369	0.979
Left femur upper neck BMD [g/cm^2^]	0.75 ± 0.2	0.814 ± 0.144	0.572
Left femur upper neck age-matched BMD [%]	92.333 ± 24.799	99.476 ± 17.394	0.734
Left femur lower neck BMD [g/cm^2^]	1.066 ± 0.139	1.075 ± 0.127	0.928
Left femur Ward’s triangle BMD [g/cm^2^]	0.781 ± 0.119	0.795 ± 0.148	0.751
Left femur Ward’s triangle age-matched BMD [%]	88.111 ± 10.925	88.286 ± 15.153	0.964
Left femur trochanter BMD [g/cm^2^]	0.726 ± 0.126	0.725 ± 0.125	0.982
Left femur trochanter age-matched BMD [%]	86.222 ± 12.143	85.333 ± 12.055	0.803
Left femur shaft BMD [g/cm^2^]	1.146 ± 0.138	12.055 ± 0.167	0.635
Left femur total BMD [g/cm^2^]	0.956 ± 0.122	0.948 ± 0.136	0.965
Left femur total age-matched BMD [%]	95.333 ± 9.152	95.048 ± 12.002	0.909
L1–L4 BMD [g/cm^2^]	1.218 ± 0.212	1.261 ± 0.181	0.476
L1–L4 age-matched BMD [%]	101.333 ± 14.361	104.1 ± 10.004	0.570
L1 BMD [g/cm^2^]	1.121 ± 0.176	1.189 ± 0.218	0.469
L1 age-matched BMD [%]	97.444 ± 12.4	101.55 ± 10.928	0.369
L2 BMD [g/cm^2^]	1.199 ± 0.252	1.262 ± 0.175	0.222
L3 age-matched BMD [%]	98 ± 16.256	103 ± 11.272	0.257
L3 BMD [g/cm^2^]	1.261 ± 0.233	1.324 ± 0.184	0.175
L3 age-matched BMD [%]	103.111 ± 15.004	107.45 ± 9.506	0.310
L4 BMD [g/cm^2^]	1.262 ± 0.233	1.259 ± 0.181	0.928
L4 age-matched BMD [%]	103.667 ± 17.493	102.45 ± 10.899	0.887
L1–L2 BMD [g/cm^2^]	1.162 ± 0.212	1.227 ± 0.189	0.319
L1–L2 age-matched BMD [%]	97.778 ± 14.087	102.5 ± 11.009	0.408
L1–L3 BMD [g/cm^2^]	1.197 ± 0.217	1.262 ± 0.186	0.205
L1–L3 age-matched BMD [%]	100.444 ± 14.293	105 ± 10.126	0.321
L2–L3 BMD [g/cm^2^]	1.232 ± 0.239	1.294 ± 0.176	0.124
L2–L3 age-matched BMD [%]	100.778 ± 15.352	105.35 ± 9.99	0.228
L2–L4 BMD [g/cm^2^]	1.245 ± 0.228	1.28 ± 0.175	0.469
L2–L4 age-matched BMD [%]	101.889 ± 15.536	104.25 ± 10.02	0.637
L3–L4 BMD [g/cm^2^]	1.263 ± 0.225	1.289 ± 0.18	0.556
L3–L4 age-matched BMD [%]	103.667 ± 15.716	104.65 ± 9.832	0.869

Notes: BMD, bone mineral density.

**Table 2:** Patient characteristics—clinical characteristics and laboratory tests. Group 1—patients with classical Ehlers-Danlos syndrome; Group 2—patients with hypermobile Ehlers-Danlos syndrome.

Characteristics	Group 1 (N = 9)	Group 2 (N = 21)	Significance of the Difference (*p*-Value)
Age [years]	39.8 ± 8.3	33.5 ± 9.0	0.045
Body weight [kg]	74 ± 20.3	68 ± 17.4	0.283
BMI [kg/m^2^]	26.7 ± 6.6	25.3 ± 6.6	0.348
Total calcium [mmol/L]	2.403 ± 0.106	2.399 ± 0.074	0.756
Inorganic phosphate [mg/dL]	3.642 ± 0.958	3.406 ± 0.443	0.449
PTH [pg/mL]	50.522 ± 16.4	39.552 ± 13.679	0.064
25-OH vitamin D [ng/mL]	22.489 ± 10.144	34.076 ± 18.011	0.022
Alkaline phosphatase [µg/L]	10.7 ± 2.46	9.029 ± 2.672	0.077
CTx [ng/mL]	0.379 ± 0.209	0.395 ± 0.181	0.789
Osteocalcin [ng/mL]	19.4 ± 7.191	21.081 ± 7.597	0.449
Sodium [mmol/L]	140.889 ± 2.759	141.619 ± 2.085	0.563
Potassium [mmol/L]	4.766 ± 0.301	4.599 ± 0.403	0.193
TSH [µIU/mL]	1.946 ± 0.975	2.187 ± 1.526	0.929
DHEA-S [µg/dL]	194.278 ± 85.494	217.086 ± 103.734	0.624
Cortisol [µg/dL]	14.756 ± 4.231	15.243 ± 6.63	0.859
History of fractures	4 (44.44%)	13 (61.905%)	0.476

Notes: BMI, body mass index; CTx, CrossLaps; DHEA-S, dehydroepiandrosterone sulfate; PTH, parathyroid hormone; TSH, thyroid-stimulating hormone; total calcium—normal range: 2.09–2.54 mmol/L; inorganic phosphate—normal range: 2.5–4.5 mg/dL; parathormone—normal range: 14.9–56.9 pg/mL; bone-specific alkaline phosphatase—normal range: 5.5–24.6 µg/L; beta-CrossLaps (CTx)—normal range: 0.148–0.967 ng/mL; osteocalcin—pre-menopausal normal range: 11–43 ng/mL; sodium—normal range: 135–145 mmol/L; potassium—normal range: 3.5–5.3 mmol/L; thyroid-stimulating hormone—normal range: 0.27–4.2 µIU/mL; dehydroepiandrosterone sulfate (DHEA-S)—normal range: 148–407 µg/dL; and cortisol—normal range: 6.02–18.4 µg/dL.

**Table 3:** Multivariate logistic regression in the assessment of the predictors of a Z-score below −2.0.

Variable	Odds Ratio, OR (95% Cl)	Significance of the Difference (*p*-Value)
Hypermobile Ehlers-Danlos syndrome	0.067 (0.0–20.927)	0.356
Age	0.975 (0.823–1.155)	0.766
Body mass index	0.947 (0.724–1.24)	0.694
Total calcium	1.622 (0.0–32,902,246.74)	0.955
Inorganic phosphate	2.523 (0.137–46.529)	0.534
Alkaline phosphatase	0.550 (0.219–1.38)	0.203
Parathormone	0.978 (0.828–1.155)	0.792
CrossLaps (CTx)	0.022 (0.0–12,177.508)	0.571
Osteocalcin	1.057 (0.789–1.417)	0.711
25-OH vitamin D	0.944 (0.816–1.093)	0.442

## Data Availability

The datasets generated and/or analyzed during the present study are available from the corresponding author on reasonable request.
